# The promise and peril of health systems

**DOI:** 10.1111/1475-6773.13595

**Published:** 2020-12-07

**Authors:** Richard Kronick

**Affiliations:** ^1^ Herbert Wertheim School of Public Health University of California San Diego La Jolla California USA

## 1 INTRODUCTION

Soon after I had the privilege of becoming Director of the Agency for Healthcare Research and Quality (AHRQ), in 2013, I remarked that I had heard innumerable times that Geisinger Health System was one of the highest performing health care systems in the United States. I said that I was perfectly willing to believe it, but that I had no way of knowing for sure. Even with AHRQ’s resources at my fingertips, and even after nearly four decades as a health services researcher, I could not say whether Geisinger was better or worse than the next health system.

My ignorance about Geisinger's performance was emblematic of a broader problem. Health care in the United States is increasingly being delivered by physicians and hospitals that are affiliated with health systems, yet we know very little about how these health systems perform, and even less about how to foster performance improvement.

The papers in this volume, written as part of the AHRQ Comparative Health System Performance initiative, expand our knowledge. What emerges is a picture of a health care landscape increasingly dominated by large systems. Guided by smart policy, that could be very good news: Large health systems have the potential to improve equity, quality, and efficiency. Otherwise, the rise of health systems could spell trouble: They could make health care more expensive, lower quality, and be less responsive to patients. In this commentary, I describe the promise and peril of health systems, highlight striking findings from this volume, and conclude with two policy recommendations.

## 2 THE PROMISE AND PERIL OF HEALTH SYSTEMS

On the optimistic side, large health systems have the potential to achieve many goals that independent practitioners or smaller medical groups cannot, including:

*Reducing disparities*
  The 15‐year difference in life expectancy at age 40 between the rich and the poor in the United States is a human tragedy and is a scandalous commentary on our society and our health care system. Even understanding that many of the causes of this inequity lie outside of the health care system, arguably one of the main goals of health care should be to reduce this disparity. Health systems have the potential to measure and focus on reducing disparities in health care and health outcomes among their patients.
*Engaging in health planning*
  In industries other than health care, a crucial challenge for organizational managers is figuring out what configuration of inputs will most efficiently produce a given quantity of high‐quality output. For example, the CEO of an automobile manufacturer needs to figure out how many workers of various types and how much and what types of machinery are needed to produce a given volume of cars. The task of figuring out what configuration of health care inputs will most efficiently and effectively improve population health outcomes could be assigned to the government, as it is in many countries. However, in the United States the government has largely stayed out of the health planning enterprise. In the vacuum, the distorted signals of a poorly functioning market and the payment rules established by Medicare, Medicaid, and commercial insurers heavily influence the number and types of health care facilities and professionals. Large health systems could, potentially, perform the function of figuring out what types of health care resources are needed to efficiently improve health outcomes for their patients, and how those resources could best be deployed (eg, primary care redesign).
*Creating a learning health system*
  Health care delivery is beset by uncertainties about what treatments work best for whom, as evidenced by substantial geographic variation in health care utilization. Clinicians and researchers at large health care systems, working with clinically rich data from electronic health records, can produce information about what works to improve outcomes that patients’ value. Large health care systems can work at understanding and reducing unwarranted variation in how health care is delivered.
*Disseminating evidence from Patient Centered Outcomes Research (PCOR)*
  Large health systems, to the extent that they can establish themselves as a trusted source of information for physicians and patients, have the potential to reduce the long lag between evidence generation and evidence adoption.

For all their potential, the increasing dominance of large health systems also creates many causes for concern. Large health systems have the potential to:

*Add layers of bureaucracy and administrative cost*
  Large systems inevitably require a layer (or multiple layers) of management and administration not needed by independent practitioners or small practices, adding costs, and potentially stifling innovation.
*Reduce competition, raise prices, and reduce responsiveness to patients*
  Large systems, if they gain dominance in local health care markets, can raise prices to private insurers. If large systems obtain market dominance, they may feel little need to be responsive to patient or provider preferences.
*Erode physician professionalism and autonomy*
  Employment by large health systems may erode physician autonomy and weaken the sense of professionalism that is a motive force behind much quality improvement in health care.
*Influence public policy*
  Large health systems have the potential to influence public policy, moving policy in directions that benefit the health systems, and not necessarily in directions that benefit patients or the general public.

## 3 NEW KNOWLEDGE ABOUT HEALTH SYSTEMS

The discussion above is largely theoretical. In 2013, when I became Director of AHRQ, we knew very little about health systems or how they functioned.

The AHRQ Comparative Health System Performance (CHSP) initiative was conceived with the goal of generating insights about health system performance, and the conditions under which health system performance improves. The initiative started with some immodest goals, including:

- Learning how to identify health systems and the physicians and hospitals that are part of those systems. It is difficult to conduct research in an area without being able to identify the subjects of that research.
- Constructing typologies of health systems and describing the activities of health systems. What are the salient dimensions on which health systems vary in organization and behavior?
- Learning how to measure the performance of health systems. In order to answer the question about whether Geisinger is really one of the highest performing health systems in the United States, we need methods of measuring system performance, both relative to other health systems, and relative to physicians and hospitals that are not part of health systems.
- Application of the measures of system performance to create a report card of health system performance. Such a report card, if accepted as valid, would likely motivate performance improvement.
- Conducting research to understand the characteristics of systems, and of the financial and competitive incentives they face, that are associated with high performance.
- Learning what tools and resources health systems need to move toward higher performance.

The papers in this volume of Health Services Research, and the more than 60 other papers that have been published with the support of CHSP funding,^1^ have made substantial progress toward achieving some of the goals of the initiative, while still leaving much work to be done.

Progress has been made in learning how to identify health systems and the physicians and hospitals that are part of those systems. The AHRQ Compendium of Health Systems is a salutary advance, using a variety of data sources to create a publicly available data resource that allows researchers to identify the physicians and hospitals that were part of each of the 637 health systems in the United States in 2016 and in 2018.^2^ We have learned from these data that more than 50% of physicians were affiliated with a health system in 2018, a striking increase from approximately 40% in 2016.^3^ I am hopeful that this data resource will jump start research on health systems by providing a mechanism that allows researchers to easily link physicians and hospitals to the health systems with which they are affiliated.

Progress has also been made in identifying salient characteristics of systems, including new contributions from articles in this volume. Progress has been made in identifying types of integration and methods of measuring them, developing methods for measuring clinical and financial integration, in understanding whether system leaders are primarily motivated by internal or external incentives as they approach quality improvement efforts, and in identifying activities that health systems engage in as they attempt to improve quality and outcomes.^4^, ^5^, ^6^, ^7^

Development of valid and reliable measures of health system performance has been more challenging, reflecting the challenges of measuring performance in health care more generally. Risk‐adjusted total cost of care, for patients attributed to physicians in a system, is a commonly used measure.^5^, ^8^, ^9^ However, with the notable exception in this volume of analysis of data from four states with All Payer Claims Data bases, most of the research analyzing total cost of care is limited to analysis of Medicare beneficiaries.^9^ To the extent that we are concerned that large health systems are able to increase prices, analysis of data on privately insured patients is needed.

Measurement of health system performance in improving quality and outcomes has relied primarily on measures developed for measuring performance of hospitals and physician groups. For example, the strong contribution in this volume analyzing whether disparities in care are any smaller for patients of physician organizations affiliated with health systems than for patients of nonaffiliated physicians provides a useful list of measures, reflecting the state of the art.^8^ This paper analyzes a set of process measures, such as breast and colon cancer screening; medication adherence measures; care coordination measures; and utilization measures for sentinel events, such as emergency department visits for ambulatory care sensitive admissions.

While good progress has been made in some areas, very little progress has been made to date on the last three bullets in my list of immodest goals above. We do not yet have a report card on health system performance that would allow comparison of performance across systems; we do not yet have much empirical evidence about the characteristics of health systems that are associated with high performance, nor of the payment and other accountability mechanisms that lead to performance improvement; and we do not yet know much about what tools and resources systems would need to facilitate improvement. Research on comparative health system performance is still in its infancy, and it is not surprising that little progress has been made to date on these important questions.

However, even in the absence of empirical evidence on many of these questions, it is no surprise that health systems have not, for the most part, engaged in reducing disparities, in reducing total cost of care, improving quality and outcomes that matter to patients, or engaged in health planning. For health systems, as for independent physicians and hospitals, there is little reward to engaging in these activities. The continued dominance of fee‐for‐service payment methods means that reducing total cost of care results in reductions in revenue. Under fee for service, hospitals are revenue centers; for systems, such as Kaiser, where virtually all of the revenue arrives through capitation, hospitals are a cost center. Similarly, primary care practices in health systems paid through fee for service typically lose money or struggle to break even; in a health system paid through capitation, strong primary care is the linchpin to success.

A health system that is able to improve quality and outcomes, to the extent that it is noticed by patients, may gain more patients, but it is difficult for patients to notice. Even more, neither patients nor third‐party payers know whether health systems have reduced disparities, and if they did, it is not clear whether systems would be rewarded for doing so. We primarily rely on the professionalism of health system managers and the clinicians in those systems to work on quality improvement. However, professionalism has largely been insufficient to overcome the cost increasing incentives created by fee‐for‐service payment, and the lack of external reward for disparity reduction or quality and outcome improvement.

## 4 TWO POLICY RECOMMENDATIONS

Patients will increasingly receive care from physicians, hospitals, and other health care providers that are affiliated with health systems. Yet we still know very little about how to improve health system performance. What to do? As a researcher, naturally my first answer is more research, particularly in the identification of high‐performing health systems, identifying the characteristics of those health systems, and developing the tools and creating the environment in which systems will move toward higher performance. As a former policy official, however, I am inpatient with the pace of research and suggest two courses of action.

First, we should continue the movement away from fee‐for‐service payment toward capitation, or, its poor cousin, shared savings approaches. Health systems cannot be expected to work hard at reducing the supply of hospital beds or procedurally oriented physicians when these resources are revenue centers as opposed to cost centers. Similarly, there is little financial reward to investing in activities that improve population health when payment is primarily on a fee‐for‐service basis.

Second, we should produce an annual report card on health system performance, with information on risk‐adjusted total cost of care, on performance on quality and outcome measures, and on disparities in care and outcomes for disadvantaged groups. Limitations in data availability, in our ability to measure what matters, and in our ability to risk‐adjust outcomes so as to not disadvantage those systems that disproportionately serve disadvantaged groups should make us wary about using this report card, at least initially, to adjust payment. But no health system will want to be at the bottom of this report card, and even without payment incentives, systems will work at improving their ranking. For example, if we measure and report on disparities in care and outcomes within health systems, it seems likely that systems will work on reducing disparities. As I and others have written elsewhere, there is great danger in using strong incentives to reward performance when the performance that can be measured is only a small subset of the performance that people care about.^10^ But especially in a world that is increasingly dominated by large health systems, absence of accountability mechanisms is also a perilous path. Much greater levels of public investment in performance measurement and improvement will be needed to capitalize on the potential that the increasing dominance of health systems makes possible.

I still can't tell you whether Geisinger is the highest performing health system in the United States. But I can tell you that health systems are growing in size, and are the dominant form of health care delivery in many communities. As researchers and policy makers, we can potentially leverage that growth to improve equity and quality, increase accountability, and lower costs. However, as the papers in this volume make clear, there is nothing magical about large health systems that lead to improvements, and concerted research and policy efforts will be needed to realize the potential.
